# Global left ventricular load in asymptomatic aortic stenosis: covariates and prognostic implication (the SEAS trial)

**DOI:** 10.1186/1476-7120-10-43

**Published:** 2012-11-05

**Authors:** Åshild E Rieck, Eva Gerdts, Mai Tone Lønnebakken, Edda Bahlmann, Giovanni Cioffi, Christa Gohlke-Bärwolf, Simon Ray, Dana Cramariuc

**Affiliations:** 1 Institute of Medicine, University of Bergen, Bergen, Norway; 2 Haukeland University Hospital, Bergen, Norway; 3 Asklepios Clinic St. Georg, Department of Cardiology, Hamburg, Germany; 4 Department of Cardiology, Villa Bianca Hospital, Trento, Italy; 5 Herz-Zentrum Bad Krozingen, Bad Krozingen, Germany; 6 Department of Cardiology, North West Heart Centre, University Hospitals of South Manchester, Manchester, UK

**Keywords:** Aortic valve stenosis, Hypertension, Valvuloarterial impedance, Prognosis, Echocardiography

## Abstract

**Introduction:**

Valvuloarterial impedance (Zva) is a measure of global (combined valvular and arterial) load opposing left ventricular (LV) ejection in aortic stenosis (AS). The present study identified covariates and tested the prognostic significance of global LV load in patients with asymptomatic AS.

**Methods:**

1418 patients with mild-moderate, asymptomatic AS in the Simvastatin Ezetimibe in Aortic Stenosis (SEAS) study were followed for a mean of 43±14 months during randomized, placebo-controlled treatment with combined simvastatin 40 mg and ezetimibe 10 mg daily. High global LV load was defined as Zva >5 mm Hg/ml/m^2^. The impact of baseline global LV load on rate of major cardiovascular (CV) events, aortic valve events and total mortality was assessed in Cox regression models reporting hazard ratio (HR) and 95% Confidence Intervals (CI).

**Results:**

High global LV load was found in 18% (n=252) of patients and associated with female gender, higher age, hypertension, more severe AS and lower ejection fraction (all p<0.05). A total of 476 major CV events, 444 aortic valve events and 132 deaths occurred during follow-up. In multivariate Cox regression analyses, high global LV load predicted higher rate of major CV events (HR 1.35 [95% CI 1.08-1.71], P=0.010) and aortic valve events (HR 1.41 [95% CI 1.12-1.79], P=0.004) independent of hypertension, LV ejection fraction, female gender, age, abnormal LV geometry and AS severity, but failed to predict mortality.

**Conclusion:**

In asymptomatic AS, assessment of global LV load adds complementary information on prognosis to that provided by hypertension or established prognosticators like AS severity and LV ejection fraction.

## Background

Patients with aortic stenosis (AS) often have hypertension, [1-5] which is associated with stiffening of the arterial tree, vascular atherosclerosis and increased incidence of ischemic cardiovascular (CV) events in AS [6,7]. The combined valvular and arterial load imposed on the left ventricle (LV) in AS can be noninvasively quantified by calculation of the valvulo-arterial impedance (Zva) [3]. High global LV load was associated with increased mortality in a previous retrospective study of patients with asymptomatic, moderate-to-severe AS [8]. These findings were confirmed in a prospective study by Lancellotti et al. in 163 patients with asymptomatic AS, demonstrating that higher global LV load predicted increased risk of developing symptoms, cardiac death and need for aortic valve replacement, independent of peak aortic jet velocity, left ventricular systolic longitudinal deformation and left atrial area index [9].

The aim of the present study was to further characterize the phenotype associated with high global LV load and prospectively evaluate if high global LV load predicted increased rate of CV events also in patients with milder AS beyond the increased risk associated with concomitant hypertension and other known prognosticators in AS like AS severity and LV ejection fraction [7,10].

## Methods

### Study population

**Figure 1:**
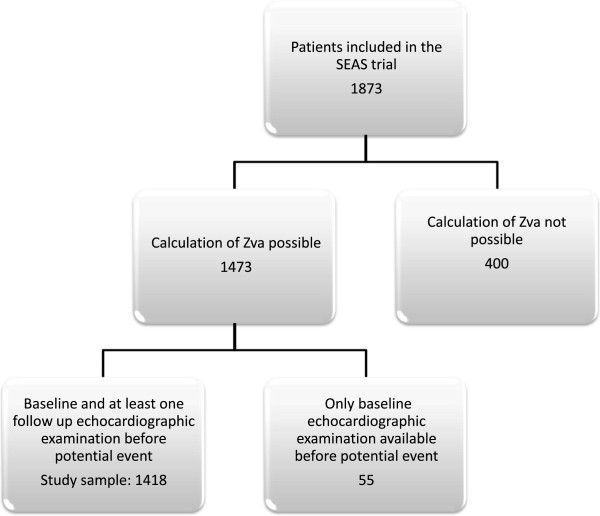
Flow chart of patient enrolment.

The methods and results of the prospective Simvastatin Ezetimibe in Aortic Stenosis (SEAS) study which tested the effect of randomized, double-blind, placebo-controlled treatment with combined simvastatin and ezetimibe on AS progression and CV morbidity and mortality in 1873 patients with initially asymptomatic, mild-to-moderate AS, have been reported [11]. All of the patients gave written informed consent, and ethical committees in all of the participating countries approved the study. The study demonstrated that lipid-lowering treatment reduced ischemic CV events in these patients, but did not reduce progression of AS or need for aortic valve replacement [12,13]. The present study population included the 1446 of the total 1873 patients in the SEAS study in whom Zva could be assessed on the baseline echocardiogram and who had at least one follow-up echocardiogram before occurrence of any study endpoint (Figure 1). Hypertension was defined as history of hypertension reported by the attending physician or elevated blood pressure at the baseline clinical visit (systolic blood pressure ≥140 mmHg and/or diastolic blood pressure ≥ 90 mmHg) [14].

### Echocardiographic measurements

Study echocardiograms were recorded at 173 SEAS study sites following a standardized protocol and sent for expert interpretation at the SEAS echocardiography core laboratory at Haukeland University Hospital. The echocardiographic protocol has been previously published [1,15,16]. All reading was performed using off-line digital workstations with Image Arena® (TomTec Imaging Systems GmbH, Unterschleissheim, Germany) software and proof-read by a single experienced reader (EG).

### Assessment of LV geometry and function

LV structure and systolic function were measured following the joined European Association of Echocardiography and American Society of Echocardiography guidelines [17]. LV mass was calculated using an autopsy validated formula [18]. LV hypertrophy was considered present when LV mass/height^2.7^ exceeded 46.7 g/m^2.7^ in females and 49.2 g/m^2.7^ in men, respectively [19,20]. Relative wall thickness was calculated as the LV posterior wall thickness/LV internal radius ratio at end-diastole, and concentric geometry was defined as relative wall thickness >0.43 [21]. Left ventricular ejection fraction was assessed by biplane Simpson’s method and considered low if <50% [22].

### Assessment of valvular and arterial disease

Severity of AS was assessed following current guidelines [10]. Energy loss index was calculated by a validated equation [23,24]. Global LV load was assessed as valvulo-arterial impedance (Zva) calculated by the method published in the paper by Briand et al. taking into account the net mean aortic gradient and thus the phenomenon of pressure recovery: Zva = (Systolic arterial pressure + Mean net aortic gradient) / (Stroke volume/body surface area) [3]. The net mean gradient was calculated as the mean aortic gradient corrected for actual pressure recovery in the individual patient [25,26]. Pressure recovery (mm Hg) was calculated at the sinotubular junction level of the aorta as 4v^2^ × 2AVA/Aa[1 – (AVA/Aa)], where v is the mean aortic jet velocity, AVA is calculated by the continuity equation and Aa is the aortic area [3]. Stroke volume was calculated by the Doppler method and indexed for body surface area [27].

### SEAS study endpoints

The primary endpoint of the prospective SEAS study was major CV events, including hospitalization for heart failure due to progression of AS, death from CV causes, aortic valve replacement, non-fatal myocardial infarction, hospitalization for unstable angina, coronary revascularization and non-hemorrhagic stroke [13]. Secondary endpoints were aortic valve related events (combined congestive heart failure attributed to progression of AS, aortic valve replacement, and death from CV causes) and ischemic CV events (combined death from CV causes, non-fatal myocardial infarction, hospitalization for unstable angina, coronary revascularization, and non-hemorrhagic stroke) analysed separately. Total mortality was a pre-specified tertiary endpoint. All endpoints were adjudicated by an independent committee blinded to study treatment.

### Statistical analyses

IBM SPSS 20.0 (SPSS, Chicago, IL) software was used for data management and analysis. The study had a statistical power of 95% to detect a 30% difference in incidence of major CV events, the primary study end-point, with a significance level of 0.01. Data are expressed as mean ± standard deviation for continuous variables and as percentages for categorical variables. High global left ventricular load was defined as Zva > 5.00 mm Hg/ml/m^2^[3,9]. Groups were compared by Students t-test or chi square test as appropriate. Covariates of higher global LV load were identified in univariate correlations and in multivariate linear regression analysis.

Kaplan-Meier curves were used to compare cumulative hazard of CV events in groups of patients who had lower vs. higher global LV load at baseline. Uni- and multivariate Cox regression analyses were used to assess the relation of baseline global LV load to major CV events, aortic valve events, ischemic CV events and total mortality. In the primary multivariate model, global LV load, randomized study treatment, hypertension, LV ejection fraction, female gender, age and abnormal LV geometry were included as covariates. In further models, conventional indices of AS severity, like aortic valve area, mean transaortic gradient and peak aortic jet velocity, were added as covariates. Results are reported as hazard ratio (HR) and 95% confidence interval (CI). A two-tailed p <0.05 was considered statistically significant both in univariate and multivariate analyses.

## Results

### Characteristics of patients with high global LV load

The main clinical and echocardiographic features of the study population are shown in Table 1. Patients with high global LV load (n = 252) included more women, patients with hypertension, were on average older and had higher blood pressure, lower systemic arterial compliance, and more severe AS than patients with normal global LV load (n = 1166) (Table 1). There were no differences in LV geometry between the two groups (P=0.65).

**Table 1:** Clinical and echocardiographic characteristics of the total study population and groups of patients with high vs. normal global left ventricular load at baseline

	**Total study population (N=1418)**	**Normal global LV load (N=1166)**	**High global LV load (N=252)**	**P value**
Age (yrs)	67±10	67±10	70±9	<0.001
Female gender (%)	39	37	45	0.015
Systolic blood pressure (mmHg)	145±20	146±20	155±21	<0.001
Diastolic blood pressure (mmHg)	83±10	82±10	86±10	<0.001
Body mass index (kg/m^2^)	26.9±4.3	26.7±4.2	27.4±4.5	0.053
Mean blood pressure (mmHg)	104±11	104±11	109±12	<0.001
Pulse pressure (mmHg)	65±18	64±18	70±19	<0.001
Hypertension (%)	83	81	93	<0.001
Heart rate (bmp)	66±11	65±11	68±12	0.001
Antihypertensive treatment (%)	57	55	60	0.078
Angiotensin Converting Enzyme Inhibitor (%)	15	15	15	0.933
Angiotensin Receptor Blocker (%)	11	11	10	0.737
Calcium Antagonist (%)	17	16	19	0.213
Beta Blocker (%)	28	27	32	0.126
Alpha Blocker	2	2	4	0.006
LV mass index (g/m^2.7^)	46.2±14.9	46.0±14.8	45.5±14.0	0.659
LV hypertrophy (%)	37	37	36	<0.001
Relative wall thickness (%)	0.36±0.09	0.36±0.08	0.36±0.10	0.769
Normal LV geometry (%)	57	55	57	0.658
Ejection fraction (%)	66±7	67±6	66±7	0.014
Low ejection fraction (%)	1	1	3	0.064
Mitral regurgitation				
Grade 1 (%)	38	39	37	0.567
Grade 2(%)	10	9	13	0.089
Grade 3 (%)	1	1	1	0.418
Aortic regurgitation				
Grade 1 (%)	43	43	45	0.550
Grade 2(%)	16	17	13	0.109
Grade 3 (%)	1	1	1	0.604
Aortic annulus (cm)	2.19±0.26	2.24±0.26	1.98±0.20	<0.001
Sinotubular aortic diameter (cm)	2.81±0.44	2.83±0.44	2.73±0.42	<0.001
Ascending aorta diameter (cm)	3.92±0.58	3.93±0.58	3.88±0.54	0.250
Peak aortic jet velocity (m/s)	3.08±0.54	3.07±0.53	3.13±0.56	0.131
Aortic valve area (cm^2^)	1.27±0.45	1.37±0.45	0.86±0.25	<0.001
Energy loss index	0.90±0.47	0.98±0.47	0.55±0.20	<0.001
Mean transaortic gradient (mmHg)	23±9	22±9	24±9	0.026
Systemic arterial compliance	0.71±0.29	0.83±0.33	0.43±0.10	<0.001
Zva (mm Hg/ml · m^2^)	4.13±1.35	3.50±0.80	5.91±0.92	NA
Total cholesterol (mmol/L)	5.73±1.02	5.70±1.03	5.86±0.96	0.024
Serum creatinine (mmol/L)	93±16	93±15	95±17	0.283
Doppler Stroke volume (ml)	85±25	91±24	58±10	<0.001
LV end-diastolic volume, by biplane Simpson (ml)	76±25	76±25	79±25	0.125
LV end-systolic volume, by biplane Simpson (ml)	43±22	42±22	47±24	0.022
Cardiac output by Doppler(L/min)	5.5±1.8	5.9±1.7	3.9±1.0	0.757
Cardiac index (L/min/m^2^)	2.9±0.9	3.1±09	2.1±0.5	0.629

LV: left ventricular.

In multivariate linear regression analysis higher global LV load at baseline was independently associated with hypertension, female gender, higher age, lower LV ejection fraction and more severe AS (Table 2).

**Table 2:** **Covariates of global left ventricular load at study baseline in multivariate linear regression analysis (multiple R**^**2**^**= 0.48, p<0.001)**

	**Beta**	**T**	**P value**
Constant		18.78	<0.001
Age (years)	0.04	2.08	0.037
Hypertension	0.157	7.67	<0.001
Female gender	0.10	4.82	<0.001
Ejection fraction (%)	−0.05	−2.63	0.009
Aortic valve area (m^2^)	−0.67	−31.76	<0.001

### Global LV load and prognosis

**Figure 2:**
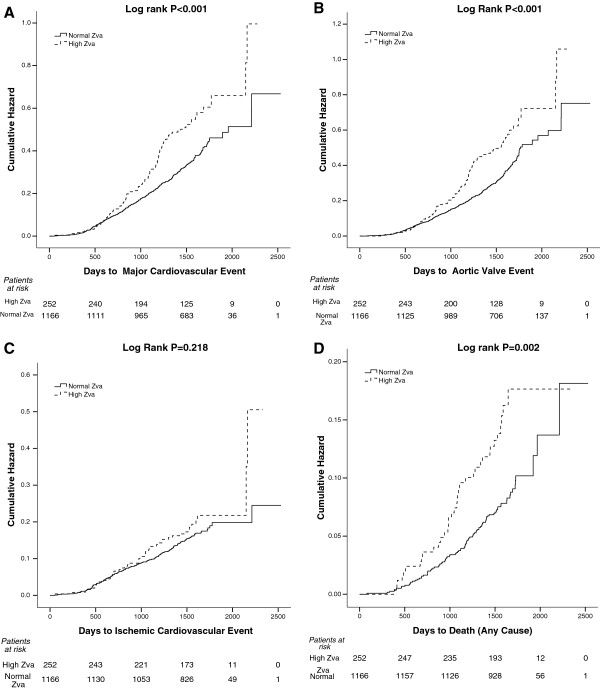
Impact of high vs. normal global left ventricular load (Zva) on cumulative hazard of major cardiovascular events (Panel A), aortic valve events (Panel B), ischemic cardiovascular events (Panel C) and total mortality (Panel D) in Kaplan Meier plots.

During a mean follow-up of 43±14 months, a total of 476 major CV events, 444 aortic valve events (387 of these were aortic valve replacements), 226 ischemic CV events and 132 deaths occurred (Table 2). Incidences of major CV events, aortic valve events and death from all causes were higher among patients with high global LV load (Table 3, Figure 2). In univariate Cox regression analyses, high baseline global LV load predicted a 49% higher rate of major CV events (95% CI 1.21-1.86%, P<0.001), a 57% higher rate of aortic valve events (95% CI 1.26-1.96%, P<0.001) as well as 83% higher rate of death from all causes (95% CI 1.25-2.69%, P=0.002). No significant association was found between global LV load and rate of ischemic CV events (Hazard Ratio 1.23, 95% CI 0.87-1.69, P=0.219). In multivariate analyses, adjusting for randomized study treatment, hypertension, LV ejection fraction, female gender, age and abnormal LV geometry, higher global LV load retained its association with higher rate of major CV events and aortic valve events, while the association with total mortality was attenuated (Table 4, primary model). Adding peak aortic jet velocity and mean aortic gradient to the models did not change the results (Table 4, secondary model). Forcing aortic valve area into this model attenuated the prognostic information provided by global LV load.

**Table 3:** Incidences of cardiovascular events in groups of patients with normal vs. high global left ventricular load

**Type of events**	**Patients with normal global LV load (N=1166)**	**Patients with high global LV load (N=252)**	**P value**
Major CV events	368(32%)	108 (43%)	0.001
Aortic valve events	340 (29%)	104 (41%)	<0.001
Ischemic CV events	180 (15%)	46 (18%)	0.268
Total mortality	96 (8%)	36 (14%)	0.003

CV: Cardiovascular.

**Table 4:** Impact of high global left ventricular load on patient outcome in multivariate Cox regression analyses

	**Primary model**		**Secondary model §**	
	**HR (95% CI) ‡**	**P**	**HR (95% CI) §**	**P**
Major CV events	1.49 (1.19-1.86)	<0.001	1.35 (1.08-1.69)	0.010
Aortic valve events	1.55 (1.23-1.95)	<0.001	1.41(1.12-1.78)	0.004
Ischemic CV events	0.99 (0.71-1.39)	0.962	0.91 (0.65-1.28)	0.589
Total Mortality	1.36 (0.91-2.02)	0.138	1.29 (0.86-1.95)	0.215

CV: cardiovascular.

‡Primary model included: Zva, randomized study treatment, hypertension, LV ejection fraction, female gender, age and abnormal LV geometry.

§ Secondary model included: all covariates in the primary model as well as peak aortic jet velocity and mean aortic gradient.

## Discussion

Concomitant hypertension is common among patients with AS, being found in up to 86% of patients in previous studies [1-5]. As recently demonstrated, hypertension in asymptomatic mild-to-moderate AS is associated with reduced arterial compliance, more subclinical atherosclerosis and increased incidence of ischemic CV events as well as a 2-fold higher mortality from all causes [7]. Our study is the first large prospective analysis of the importance of evaluating combined LV load from valvular and arterial disease by use of non-invasive valvulo-arterial impedance in mild to moderate asymptomatic AS. In particular, the current results contribute to phenotypic characterization of patients with high global LV load in milder degrees of AS, as well as demonstrating the impact of global LV load in prediction of major CV events beyond that provided by presence of hypertension and other well established prognosticators in asymptomatic AS like AS severity and LV ejection fraction [7,10].

### Predictors of high global LV load

The present results add to previous knowledge by demonstrating that also in milder, asymptomatic AS, high global LV load is associated with a high risk phenotype including female gender, older age, concomitant hypertension and reduced LV systolic function, independent of an association with more severe AS by conventional measures like peak aortic jet velocity, mean aortic gradient or aortic valve area. Our finding that older age was associated with higher global LV load is consistent with the consequences of vascular aging [28]. Physiological aging is indeed associated with both increased vascular and ventricular stiffness [29,30]. The pathophysiological foundation for this finding is uncertain, but multiple mechanisms have been proposed, including reduced endothelial function, modulation of collagen, neurohumoral signaling and vascular remodeling [31].

Consistent with previous reports, [5] hypertension was a frequent finding among SEAS patients, and associated with reduced arterial compliance and higher global LV load [7]. The association between higher global LV load and female gender is in agreement with previous findings by Gatzka et al. showing that the observed increased arterial stiffening in women is independent of posture [32].

The negative association between global LV load and LV ejection fraction is in line with previous studies reporting increased global LV load to be associated with reduced LV systolic function assessed by midwall shortening or longitudinal strain [33-35]. In treated hypertensive patients, lower LV systolic function has been associated with presence of subclinical coronary artery disease [28]. Of note, 57% of hypertensive patients in the present study population were treated. Our findings suggest that among patients with milder, asymptomatic AS, the phenotype associated with increased global LV load is typically that of an elderly, hypertensive woman with reduced LV systolic function.

### Global LV load and prognosis in AS

Confirming our hypothesis, global LV load predicted an increased rate of major CV events, in particular aortic valve events, independent of hypertension. As previously reported from the SEAS study, concomitant hypertension primarily predicted increased risk of ischemic CV events and mortality [7]. Of note, high global LV load predicted a statistically significant 49% increased rate of major CV events and a 55% increased rate of aortic valve events independent of other features of the high global LV load phenotype, including higher age, female gender, concomitant hypertension, and LV ejection fraction as well as abnormal LV geometry [10,12]. While the association with these well-known prognostic factors explained the increased mortality attributed to high global LV load in univariate analysis, also after further adjustment for different measures of AS severity, higher global LV load independently predicted a 35% higher rate of major CV events and a 41% increased rate of aortic valve events. These findings suggest that global LV load brings complementary prognostic information in patients with mild to moderate asymptomatic AS without otherwise known CV disease or diabetes.

Our findings expand observations from a retrospective study by Hachicha et al. in 544 patients with asymptomatic moderate AS [8]. In their study, a valvuloarterial impedance > 3.5 mmHg/ml/m^2^ predicted increased 4-year mortality, while the present study defined high global LV load as valvuloarterial impedance >5.00 mmHg/ml/m^2^. Of note, the present results add to the finding by Lancellotti et al. from a prospective study in 163 patients with asymptomatic, moderate to severe AS, that higher global LV load predict rate of major CV events independent of peak aortic jet velocity, while longitudinal deformation and left atrial area index were not assessed in the present study [9]. Furthermore, our findings expand the results from a small study by Zito et al. in 52 patients with severe asymptomatic AS and normal LV ejection fraction reporting that combined increased global LV load and reduced global longitudinal speckle strain were the best predictors of combined development of symptoms, aortic valve replacement and death [35]. In contrast, no improvement in risk prediction by global LV load was demonstrated in a multicentre study by Levy et al. in patients with low ejection fraction, low gradient severe, symptomatic AS [36,37].

### Study limitations

It has been suggested that calculating global LV load using central systolic blood pressure might yield better prediction of adverse outcome. Central blood pressure was not recorded in the SEAS trial. However, the use of central instead of brachial aortic blood pressure did not increment the predictive ability of global LV load in a recent publication [33].

The present study was not designed to assess the effect of different types of medication on the progression of global LV load. 68% of hypertensive SEAS patients were on blood pressure-lowering medication. However, at baseline, no difference in use of different classes of antihypertensive agents was found between patients with lower vs. higher global LV load. Although the study had high power to detect a difference in incidence of major CV events, including total mortality, the study did not have statistical power to detect the observed 20% difference in ischemic CV event incidence between the groups. Thus, a type 2 error cannot be excluded for the lack of association between high global LV load and rate of ischemic CV events in the present study.

## Abbreviations

AS: Aortic valve stenosis; CV: Cardiovascular; LV: Left ventricle; SEAS: Simvastatin and Ezetimibe in Aortic Stenosis; Zva: Valvulo-arterial impedance; CI: Confidence Interval.

## Competing interests

The authors declare that they have no competing interests.

## Authors’ contributions

ÅER, EG and DC designed the study, participated in data collection, performed the statistical analysis and drafted the manuscript. EB participated in the data collection. EB MTL CGB GC and SR participated in revisions of the manuscript. All authors read and approved the final manuscript.
